# Structure and Composition of Prey Communities Associated with Malaysian Mahseer, *Tor tambra* Diet Based on DNA Metabarcoding: Implication for Conservation

**DOI:** 10.21315/tlsr2025.36.3.2

**Published:** 2025-10-31

**Authors:** Nur Farhana Mohd Yusoff, Shukor Md Nor, Shairah Abdul Razak

**Affiliations:** 1 Genetics Program, Faculty of Science and Technology, Universiti Kebangsaan Malaysia, 43600 UKM Bangi, Selangor, Malaysia; 2 Department of Biological Science and Biotechnology, Faculty of Science and Technology, Universiti Kebangsaan Malaysia, 43600 UKM Bangi, Selangor, Malaysia; 3 Department of Applied Physics, Faculty of Science and Technology, Universiti Kebangsaan Malaysia, 43600 UKM Bangi, Selangor, Malaysia

**Keywords:** DNA Metabarcoding, Diet Composition, Freshwater Fish, Mahseer, Feeding Ecology, Metabarkod DNA, Komposisi Diet, Ikan Air Tawar, Kelah, Ekologi Pemakanan

## Abstract

Quantifying dietary composition is crucial for basic ecological research and to formulate conservation management. For predatory fishes, DNA-metabarcoding can yield more accurate estimates than conventional morphological-based analyses. In Southeast Asia, Malaysian mahseers are experiencing declines in the wild due to their commercial and aesthetic value. Current practice in artificial propagation for mahseer has yet to fulfil high market demand due to constraints in dietary formulations that affect fish fertility and optimal growth. Here we applied cytochrome oxidase I (COI) metabarcoding with one species of Malaysian mahseer, Tor tambra or ‘kelah’, to characterise their food assimilation and feeding habits from seven different locations of wild and farm origins. Prey DNA sequences were grouped into 54 taxonomic groups at the species level. The diet comprised four predominant classes: Insecta, Actinopterygii, Arachnida and Aconoidasida. Representative taxa from each class were detected in wild and farmed fish. However, less than a quarter of the total taxa overlapped between both fish origins. Non-metric multidimensional scaling (NMDS) indicated greater prey taxonomic diversity and composition in wild fish across different rivers compared to those in farmed fish (PERMANOVA, pseudo-F = 1.959, *p* < 0.05). Our findings suggest that prey availability from the surrounding play roles in determining the fish food composition and dietary overlap. Expanding dietary analyses could offer ways to optimise diet for cultured fish as one of strategies to reduce fishing pressure on wild populations.

## Abstract (fr)

**Abstrak:**

Pengukuran komposisi diet adalah penting untuk penyelidikan ekologi asas dan sebagai panduan pengurusan konservasi. Bagi ikan pemangsa, metabarkod-DNA boleh memberikan anggaran yang lebih tepat berbanding dengan analisis konvensional berasaskan morfologi. Di Asia Tenggara, mahseer Malaysia, Tor tambra terutama ikan liar semakin berkurang disebabkan nilai komersial dan estetika. Amalan semasa dalam pembiakan buatan untuk mahseer masih belum mampu memenuhi permintaan pasaran yang tinggi disebabkan kekangan dalam formulasi diet yang mempengaruhi kesuburan dan pertumbuhan optimum ikan. Di sini, kami menggunakan kaedah metabarkod sitokrom oksida I (COI) dalam mahseer Malaysia atau ‘kelah’ untuk mencirikan diet dan tabiat pemakanan ikan dari tujuh lokasi berbeza berasal dari kawasan liar dan kolam ternakan. Jujukan DNA mangsa ikan telah dikelompokkan kepada 54 kumpulan taksonomi berdasarkan pengenalpastian pada tahap taksonomi yang paling rendah. Diet terdiri daripada empat kelas utama: Insecta, Actinopterygii, Arachnida dan Aconoidasida. Wakil taksa dari setiap kelas dikesan dalam ikan liar dan ikan ternakan. Namun, kurang daripada satu perempat daripada jumlah taksa bertindihan antara kedua-dua kumpulan ikan. Penilaian dimensi berbilang bukan metrik (NMDS) menunjukkan kepelbagaian taksonomi dan komposisi mangsa yang lebih pelbagai dalam ikan liar merentas sungai berbanding dengan ikan dari kolam ternakan (PERMANOVA, pseudo-F = 1.959, p < 0.05). Penemuan kami mencadangkan bahawa ketersediaan mangsa dari persekitaran memainkan peranan dalam menentukan komposisi makanan ikan dan pertindihan diet. Analisis diet lanjutan boleh menawarkan penyelesaian konservasi bagi mengoptimumkan diet untuk ikan yang diternak sebagai salah satu strategi untuk mengurangkan tekanan tangkapan terhadap ikan populasi liar.

**HIGHLIGHTS**

- *Tor tambra*’s diet comprises at least 54 diverse prey species, predominantly from the classes Insecta, Actinopterygii, Arachnida, Aconoidasida, Ostracoda and Malacostraca, with other aquatic taxa contributing in smaller proportions.
- Beta diversity analysis revealed significant differences in fish prey communities across locations. Farmed mahseer showed similar, clustered prey profiles, while wild individuals had more diverse and distinct prey compositions.
- High taxonomic resolution of DNA metabarcoding highlight the importance of prey composition in surrounding environment and freshwater habitats for the thriving and long-term sustainability of Malaysian mahseer populations.

## INTRODUCTION

Planning efficient conservation programs requires an understanding of the feeding habits and interactions among species since feed not only serves as a source of nutrients and energy for growth, reproduction and other physiological processes but also has substantial impacts on fish abundance, migration and dispersal (Balık *et al*. 2003; Nyunja *et al*. 2002). Like many other organisms, fish feeding habits can reveal the influence of habitat type or structure (marine or freshwater), prey abundance and diversity, prey availability and predator food preference toward prey-predator as well as the competition interactions among the freshwater organisms (Hixon & Beets 1993; Paine 1992).

From the aquaculture perspective, the knowledge on the diet preference of fish species from the wild can contribute to the development of food formulation that fulfills fish nutritional demands for both conservation and economic purposes. Fish nutrition is crucial since feed represents approximately 50% of variable production costs and influence in the production of healthy and high-quality products (Pailan & Biswas 2022). The demand for fish is also increasing and will continue to surge following the rise in living standards across many developing countries and the increase in world population (Suresh 2018). Subsequently, the venture into growth-related aspects, such as dietary composition and species-specific diet formulation, is necessary for efficient fish farming, especially for slower-growing fish like mahseer.

Mahseer (Tor) of the Cyprinidae family is a type of enigmatic, highly diverse, yet highly threatened freshwater fish that can be found in rapidly-flowing waters with rocky bottoms of Asia and Southeast Asia (Ng 2004; Nguyen *et al*. 2008; Froese & Pauly 2022; Kottelat 2013; Pinder *et al*. 2019; Walton *et al*. 2017). *Tor tambra*, one of the Malaysian mahseer species, is widely distributed in aquaculture and fisheries, primarily targeted for human consumption (Kottelat 2013). Locally, it is known as Kelah in Peninsular Malaysia, Pelian in Sabah (Inger & Chin 1962; Mohsin & Ambak 1991), and Jurung in Indonesia (Jaafar *et al*. 2021). Besides *T. tambra*, two other Tor species are found in Malaysian freshwater bodies: *T. tambroides*, also called Empurau or Semah in Sarawak and *T. dourenensis* (Ng 2004). However, the current knowledge only indicates the presence of *T. tambra* and *T. tambroides* in the Peninsular Malaysia riverine (Jaafar *et al*. 2021, Lau *et al*. 2021), while the species separation remains a subject of debate (Pinder *et al*. 2019; Walton *et al*. 2017, Lim *et al*. 2021).

The high demand for mahseer and constant threat by anthropogenic activities, such as overfishing, hydropower dam construction, degradation, fragmentation and loss of habitats, subsequently caused a rapid decline in the wild population (Chong *et al*. 2010; Lau *et al*. 2021; Muchlisin *et al*. 2022; Pinder *et al*. 2015; Raghavan *et al*. 2011). To meet the high demand for human consumption, mahseer is heavily cultured due to its high nutritional value and unique flesh taste. However, cultured mahseer is commonly fed with food pellets that are developed for general temperate freshwater species like tilapia, carp and catfish to reduce fish feeding costs (Dani 2017). Such feeding practice combined with unsuitable diet formulation influences the captive fish’s fertility and fecundity alongside their optimal growth and susceptibility toward infection (Assan *et al*. 2021; Gonzales 2012; Jobling 2012; Lim *et al*. 2015). This is further exacerbated by the inadequacy of their taxonomy, feeding habits, autoecology, distribution and population status, which impose ensuing challenges toward preservation and conservation efforts (Pinder *et al*. 2015; Pinder & Raghavan 2013; Walton *et al*. 2017).

The identification dietary analysis using conventional methods is hindered by prey condition (either crushed or fully digested) and exhibits similar morphological appearance that can lead to inaccuracy in prey identification due to lower taxonomic resolution (Baker *et al*. 2014; Dawson *et al*. 2021). A more accurate, cost-effective and non-invasive method called DNA metabarcoding recently been developed to identify the diet preference of a studied species. This molecular-based method combines DNA barcoding with next-generation sequencing (NGS). The method involves amplifying prey DNA extracted from gut content using gene-specific universal primers (De Sousa *et al*. 2019). The prey organisms are then identified to the lowest possible taxonomic level when the prey DNA sequences matched with sequences in DNA databases, such as GenBank, Barcode of Life Data (BOLD) System, and DNA Databank of Japan (DDBJ) (Deagle *et al*. 2014; Kress *et al*. 2015; Bolyen *et al*. 2019). This approach has been widely used as an alternative method to study the dietary analysis of various organisms, such as seahorses (Lazic *et al*. 2021), cats (Forin-Wiart *et al*. 2018), bats (Hemprich-Bennett *et al*. 2021), penguins (Cavallo *et al*. 2018), beetles (Ammann *et al*. 2020), wood mice (Ozaki *et al*. 2018), and Europe hake fish (Riccioni *et al*. 2022).

The present work utilised cytochrome oxidase I (COI) amplicon-based DNA metabarcoding to examine the diet of mahseer in Peninsular Malaysia using the NGS method. It aims to characterise the prey communities’ composition from the gut content in both wild and cultured mahseer from various locations in Peninsular Malaysia. Findings pertaining to the feeding ecology of the *T. tambra* will offer valuable insights in improving mahseer fishery management and development as well as in fostering efficient conservation initiatives.

## MATERIALS AND METHODS

### Studied Fish and Sampling Locations

Mahseer samples for this study were collected from five natural tributaries (representing wild samples) and three artificial estuaries (representing cultured fish samples) in Peninsular Malaysia. The sampling process was done from December 2018 to August 2020 and assisted by several fishermen using a net or fishing rod (Table 1). A total of 13 wild fishes with body length between 23.5 cm to 46.0 cm and body weight between 125.0 g to 1060.0 g were gathered from the Tiang River, Royal Belum, Perak (ROB), Keniam River, Taman Negara, Pahang (TMN), Puah River, Terengganu (EMP) and Kuala Selangor, Selangor (KSL).

Each riverine system has different conditions concerning the degree of anthropogenic activities near the surrounding environment. ROB and TMN are gazetted under official government jurisdiction, with access restricted to nearby indigenous communities, researchers and licensed anglers, and thus serve only as a limited source of water and food. The well-maintained and highly protected location, situated deep in the tropical jungle, ensures a steady growth of the mahseer population. Meanwhile, EMP has been affected by dam construction over the last decade (Abu Bakar 2020), which has resulted in minimal interaction between humans and local species. Finally, KSL has the highest human activities since there are several commercial fisheries located near the river.

For farmed fish, eight samples of cultured fish were collected by personnel from several fisheries agencies, namely the Fisheries Research Institute Glami Lemi, Negeri Sembilan (FRI), Agro-Biotechnologies Institute Serdang, Selangor (ABI) and WildRec, Empangan Puah, Terengganu (TNB). The body length of these cultured fishes ranged from 22.9 cm to 41.3 cm with body weight between 138.0 g to 559.0 g. The body sizes of cultured samples gathered from all three locations were chosen randomly; however, efforts were made to match their body lengths and weights with samples collected from the wild. The fish samples were maintained in an artificial environment (cement or polycarbonate tank) that was suitable for fish growth and these criteria varied according to the fisheries. The fish from ABI were progenies of the brood stocks, which were initially captured from wild habitat and acclimatised to groundwater before being allowed to breed in a hatchery. They were fed twice daily with pellets containing 42% protein and 6% lipid. Meanwhile, the fish from FRI and TNB were reared in fish tanks with water drawn from a local stream to imitate natural freshwater conditions like temperature, pH and oxygen content. Their feeds consisted of commercial carp fish pellets. The animal care and all experimental procedures in this study were approved by the Universiti Kebangsaan Malaysia Animal Ethics Committee (FST/2019/SHAIRAH/20-MAR./992-MAR.-2019-AUG.-2022).

### Sample Preparation and DNA Extraction

Fish individuals were first anesthetised through immersion in ice prior to being dissected aseptically using sterile forceps, knives and scissors. The dissection process began by cutting the fish’s ventral region longitudinally from the back of the posterior gills to the anus fins. Two horizontal cuts were made at either end of the first cut to open the coelom and expose the viscera. The entire gastrointestinal tract (GIT), starting from the esophagus to the anus, was separated from the other viscera organs and removed from the fish body. The whole excised GITs were immediately placed into 70% ethanol as temporary storage medium and transported on ice back to the laboratory, where they were stored at −20°C until DNA isolation.

To obtain digesta samples (tissue and cell fragments from the preys), the fish’s GIT was rinsed with sterile 70% ethanol, then cut open using sterile dissecting scissors and resuspend in the same ethanol solution to maximise gut content recovery. A 50 mL aliquot of the GIT content, along with 70% ethanol, were transferred to a 50 mL centrifugal tube and centrifuged at an RCF speed of 15,000 xg for 15 min at 4°C (Ultracentrifuge, Sorvall). The GIT pellets were ground using mortar and pestle in the presence of liquid nitrogen to ensure sample homogenisation. Approximately 150 mg to 250 mg of GIT pellets from each sample were used for DNA extraction, representing the entire diet of the respective samples. DNA was extracted using a slightly modified version of the QIAamp PowerFecal Pro DNA Kit (QIAGEN, Hilden, Germany) protocol. The mixture of GIT content and Solution CD1 in the PowerBead Pro Tube was vortexed horizontally for 15 min at maximum speed. After the DNA elution, the DNA concentration was quantified with Thermo Scientific™ NanoDrop™ One/OneC Microvolume UV-Vis Spectrophotometer (ThermoFisher Scientific, Wisconsin, USA). All samples were diluted using Solution C6 from the QIAamp PowerFecal Pro DNA Kit to a standard concentration of 20 ng/μL of DNA.

### PCR Validation, Library Preparation and DNA Sequencing

PCR amplification of the COI region was conducted in 12.5 μL reactions using 80 ng of template DNA, 0.25 μL of each primer, 6.25 μL of TopTaq Master Mix and 1.25 μL of coral red stain (TopTaq Master Mix Kit, QIAGEN) to validate the presence of intact DNA. The Folmer barcoding region for mitochondrial COI (~316 bp) was amplified with freshwater macroinvertebrate primers BF1 (5′-ACWGGWTGRACWGTNTAYCC-3′) and BR2 (5′-TCDGGRTGNCCRAARAAYCA-3′) (Elbrecht & Leese 2017). Reactions were amplified starting with an initial 3 min incubation at 94°C, followed by 40 cycles of 94°C for 30 sec, 50°C for 30 sec, and 65°C for 2 min, before a final elongation step of 65°C for 5 min. The primers were chosen due to their relatively short target sequence (~316 bp), the large taxonomic breadth, and because the selected macroinvertebrate-specific primer pair displayed the best and highest consistent taxa detection rates among the BF/BR primer pairs (Elbrecht & Leese 2017). PCR products were analysed using 1.5% agarose gel electrophoresis producing ~316 bp of amplified COI segments.

Subsequently, generation sequencing library preparations and Illumina MiSeq sequencing were conducted at GENEWIZ, Inc. (Suzhou, China). The DNA samples were quantified using a Qubit 2.0 Fluorometer (Invitrogen, Carlsbad, CA, USA). Approximately 50 ng to 100 ng of DNA was used to generate amplicons using a similar panel of primers sequence BF1/BR2 designed by GENEWIZ (GENEWIZ, Inc., South Plainfield, NJ, USA). Besides the COI target-specific sequences, the primers also contained adaptor sequences that allowed a uniform amplification of the library with high complexity readiness for downstream NGS sequencing on the Illumina Miseq platform. The DNA libraries were later validated by Agilent 2100 Bioanalyzer (Agilent Technologies, Palo Alto, CA, USA), quantified by Qubit 2.0 Fluorometer (Invitrogen, Carlsbad, CA), and multiplexed and loaded on an Illumina MiSeq instrument according to the manufacturer’s instructions (Illumina, San Diego, CA, USA). Sequencing was performed using a 2 × 250 paired-end (PE) configuration while image analysis and base calling were conducted by the MiSeq Control Software (MCS) embedded in the MiSeq instrument.

### DNA Sequence Processing

The QIIME version 1.9.1 data analysis package (Caporaso *et al*. 2010) was used to process raw COI reads. The forward and reverse reads were joined and assigned to samples based on barcode and truncated by cutting the barcode and primer sequence using Cutadapt ver. 1.9.1. Quality filtering on joined sequences was performed and any sequence that did not fulfill the following criteria was discarded: sequence length < 200bp, no ambiguous bases, and mean quality score (Q) ≥ 20. The detection and removal of chimeric sequences were achieved using the UCHIME algorithm. All effective sequences were used in the final analysis whereby they were grouped into operational taxonomic units (OTUs) using the VSEARCH (1.9.6) clustering programme against the non-redundant nucleotide NT/NR (NCBI) database pre-clustered at 97% sequence identity. The NT/NR database was then used to assign taxonomic categories to all OTUs at a confidence threshold of 0.8. The decision to use the NT/NR database was prompted by the fact that it has taxonomic categories that are predicted to the species level. Meanwhile, the Barcode of Life Data (BOLD) retrieved from the Application Programming Interface (API) (see Porter & Hajibabaei 2018) and the DNA Databank of Japan (DDBJ) databases were employed for further identification of any OTUs that were previously unidentified through matches with NCBI databases. All sequences originating from the fish host were removed from further analyses and the remaining sequences were standardised so that the sequence of each taxon was represented as a proportion of all prey sequences in the sample.

### Prey Taxa Composition and Ecological Statistical Analysis

Three metrics were measured to determine the dietary preference and prey taxa composition of *T. tambra*, namely the percentage frequency of occurrence (FOO), weighted percentage of occurrence (wPOO), and relative read abundance (RRA) (Collins *et al*. 2007; Deagle *et al*. 2019). FOO represents the percentage of fish individuals that contain taxon within their prey community while wPOO is a rescaled version of FOO that assigns weights to all occurrences based on the number of food items in the sample (Deagle *et al*. 2019). Whilst both FOO and wPOO heavily rely on the presence of taxa data, RRA utilises sequence count data by presenting the percentage of sequence counts for a taxon relative to the total sequence counts detected for all taxa in the prey community across all samples. It gives an equal weighting or importance to the overall taxonomic abundance by averaging the RRA values of each sample (Deagle *et al*. 2019).

Multivariate statistical analyses were performed using R package 3.4.1 by utilising the presence and abundance of prey taxa data in the mahseer community. Sequences were rarefied prior to the calculation of alpha and beta diversity statistics. Alpha diversity indexes were calculated using *phyloseq* package from rarefied samples based on three diversity measures, namely Shannon index, Chao1 index and the observed number of OTUs (McMurdie & Holmes 2013). The prey diversity of mahseer between the wild and farmed groups was compared through the boxplot of alpha diversity indices that were generated using ggplot package. Beta diversity was estimated using R package vegan (Oksanen *et al*. 2017) based on Bray-Curtis (BC) dissimilarity distance.

Additionally, visualisation via a two-dimensional (2D) ordination plot was done based on non-metric multidimensional scaling (NMDS) in R using metaMDS command to depict differences in the prey community composition of T. tambra in terms of locations and populations. In NMDS analysis, coordinates of an object (each fish individual) are plotted into similarity and dissimilarities sets based on rank without any emphasis on the magnitude of similarities or dissimilarities. Fish individuals will be plotted at the closer distance when they share a greater amount of similarities in prey community composition harbored from their gut. Conversely, they will be plotted further away when they share less similarity based on BC measures. Following the visualisation using an ordination plot, the Permutational Multivariate Analysis of Variance (PERMANOVA) was performed with the adonis function to analyse the significance of location and population heterogeneity on the prey taxonomic and structural communities obtained from the mahseer’s stomach contents. Subsequently, the p-value of ≤ 0.05 indicated significant differences and the significance was further investigated using the One-Way Analysis of Similarity (ANOSIM) test (Clarke 1993; Shepard 1962). The purpose was to determine the individual taxa contributing to overall dissimilarity in the mahseer prey community between the wild and farmed populations.

## RESULTS

### Sequencing Results

The genomic DNA of prey items from the Tor samples were successfully amplified based on targeted mitochondrial COI region, yielding over 1,500,000 reads with an average length of ~350 bp. Paired-end sequencing generated between 55,300 and 102,614 reads across all 22 samples. After chimera removal, the read count was reduced to a range of 39,587 to 87,022. Further filtering was applied to remove singletons, and sequences were ultimately rarified to 37,573 reads per sample, based on the lowest count sample. A total of 144 OTUs were identified as potential prey items based on alignment to reference sequences on GenBank. A low percentage of prey sequences (6.33%) was detected relative to the overall sequence reads in correspondence to the fish host DNA that was taxonomically identified as *T. tambra*. Subsequently, the prey sequences of 126 out of the 144 OTUs were grouped into 43 prey items based on the lowest taxonomic classification (species level) that could be determined from the GenBank DNA database. Attempts to identify unclassified taxa from the remaining 18 OTUs were achieved using the DDBJ and BOLD Systems databases, leading to the identification of 11 additional prey items. As a result, a total of 54 prey items were classified at the species level.

### Occurrence and Detection of Prey Taxa in Wild and Farmed Mahseer

**FIGURE 1:**
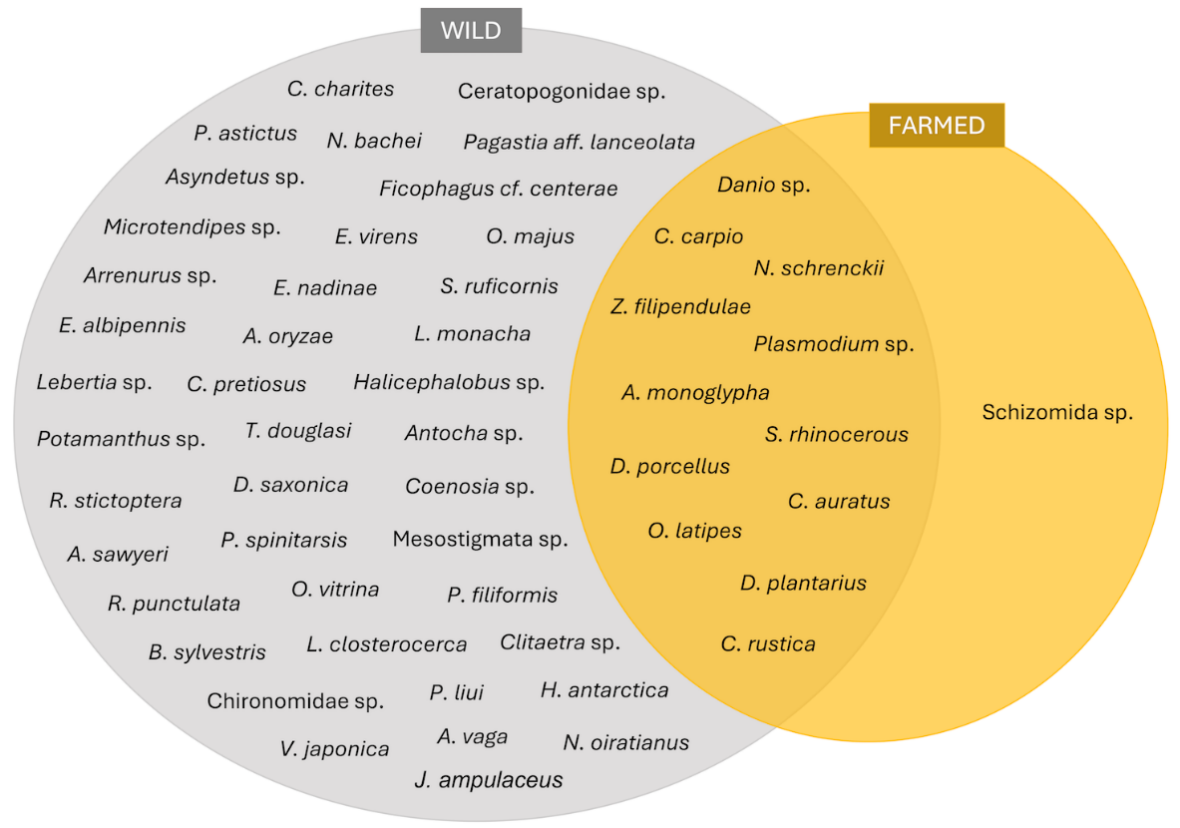
Venn diagram showing all 54 species taxa detected across samples from wild vs. farmed origins.

The quantification of FOO and wPOO offered an overview of the presence of taxa in the sample prey community eaten by mahseer. Overall, 12 classes of taxa (54 species) were found in the gut digesta of wild mahseer compared to only 4 classes (13 species) in the cultured fish collected from farm. This denotes a higher number of prey communities in wild fish than in cultured populations (Fig. 1, Supplementary Table S1).

Of these 12 classes, three classes Insecta, Actinopterygii and Aconoidasida are frequently detected in fish gut samples from wild and farmed origins. For wild fish, total four taxa along with Arachnida class comprises 49.4% wPOO of all prey taxa obtained from the wild GI. Meanwhile, the remaining 50.6% wPOO was filled by eight classes – Ostracoda, Malacostraca, Bdelloidea, Monogononta, Hydrozoa, Phaeophyceae, Chromadorea and Xanthophyceae – with FOO ranging from 1.3% to 15.9%. However, three classes (Insecta, Actinopterygii and Aconoidasida) contributed 74.3% to the cultured wPOO dataset while the remaining 25.7% wPOO from the cultured population was filled by exclusively one class, namely Arachnida (Supplementary Table S1).

**FIGURE 2:**
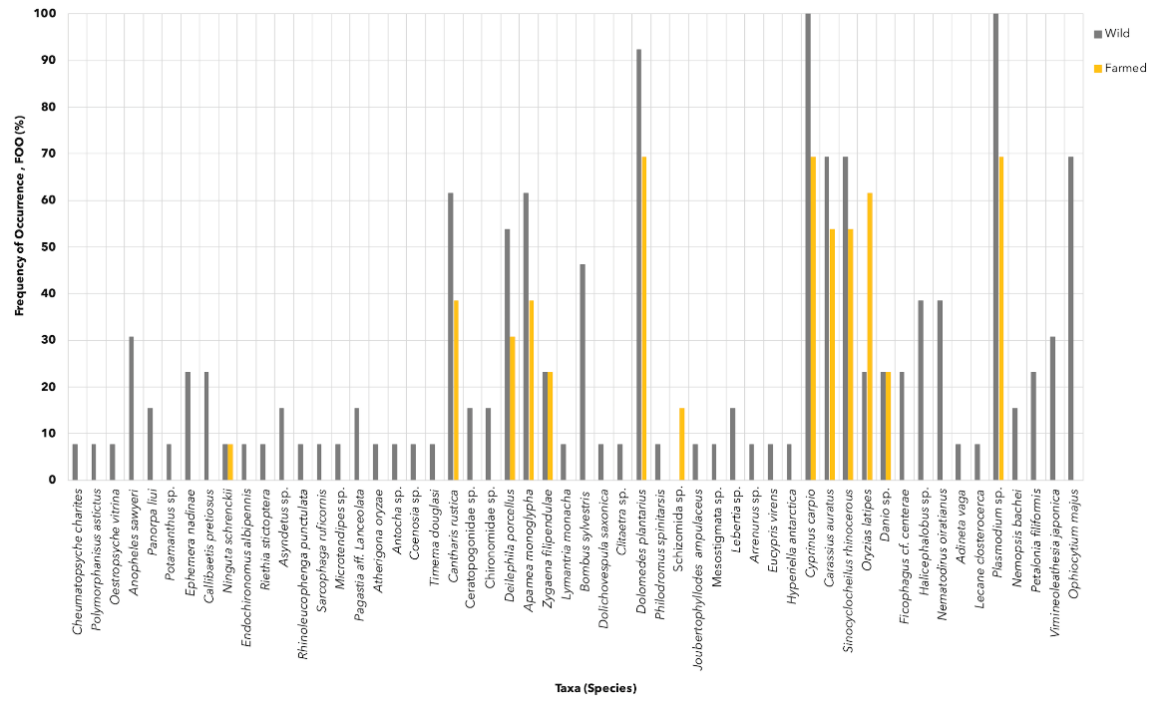
The percentage frequency of occurrence (FOO) of prey community at the lowest taxonomic level (species) in wild (denoted with grey colour) and farmed (denoted with yellow colour) mahseer.

The presence of *Cyprinus carpio* (class Actinopterygii), *Plasmodium* sp. (Aconoidasida) and *Dolomedes plantarius* (Insecta) was frequently detected in the gut digesta of both wild and farmed fish populations. Almost all wild fish individuals harbored these taxa in their guts, with FOO values ranging between 92.3% to 100.0%. Meanwhile, more than half of the farmed GI samples contained the same three taxa with the FOO value of 69.2% (Fig. 2, Supplementary Tables 1 and 2). Only 53 taxa species were present in the gut digesta of wild mahseer, with 12 of those were also detected in the gut digesta of farmed mahseer. One taxa (*Schizomida* sp.) was exclusively present in farmed fish only with an FOO of 15.4% (Figs. 1 and 2, Supplementary Table S2). A combination of 26 rare taxa was detected in some wild fish datasets (FOO < 10%). All taxa present in both wild and farmed datasets were either equally or more frequently occurred in wild fish than farmed, except for Oryzias latipes (O. latipes), which uniquely exhibited almost three times higher FOO value (61.5%) than in the wild gut samples (23.1%) (Fig. 2, Supplementary Table S2).

### Relative Read Abundance of Prey Taxa in Wild and Farmed Mahseer

**FIGURE 3:**
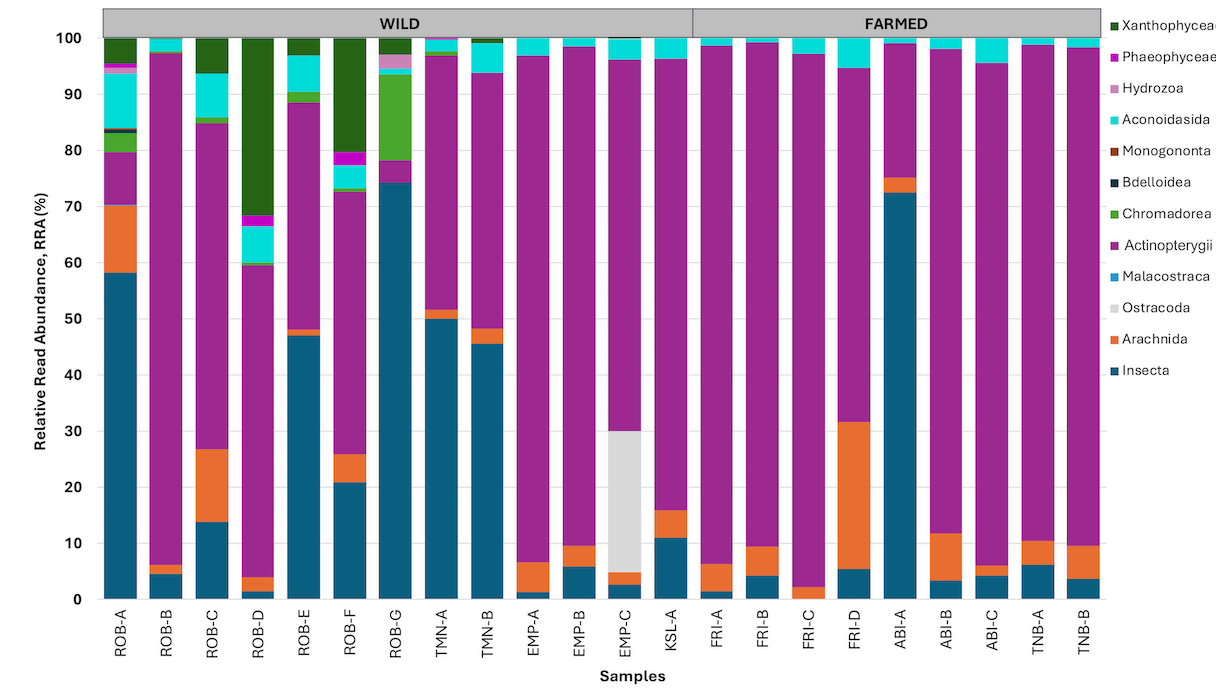
Stacked bar graph represents the relative read abundance (RRA) of prey community at the class level in wild and farmed mahseer samples. Fish individual from each sampling location were arranged alphabetically.

**FIGURE 4:**
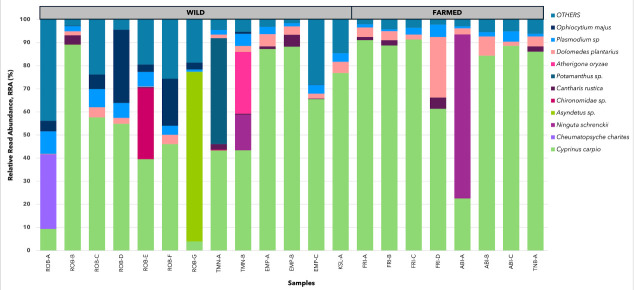
Stacked bar graph showing the 10 species with the highest relative read abundance (RRA) in the prey community of 22 GIT samples from both wild and farmed mahseer populations. Only the top ten species with their respective abundances are shown in the figure; other species are combined as “Others.” The species categorised as “Others” are shown in Supplementary Table 2. FRI-B serves as the control sample, where its DNA is extracted from the GIT of FRI-A.

Apart from detecting the presence of prey communities inside the fish digesta, this study later analysed prey taxa by their abundance (Figs. 3 and 4). For all four wild fish populations (ROB, TMN, EMP and KSL), Insecta dominated nearly half of the prey communities harbored from GI with a average relative abundance of 48.6% (Fig. 3). Meanwhile, Actinopterygii recorded 22.7% of prey abundance, followed by Arachnida (10.0%) and Aconoidasida (8.3%). Other classes of taxa had less than 0.5% abundance (Bdelloidea, Monogononta and Malacostraca) as they were found in fish individual ROB-A only. Finally, the Ostracoda class was only found in EMP-C fish individuals (RRA = 0.52%).

Unlike the wild population, prey community in the GI fish samples of the farmed populations (FRI, ABI and TNB) dominated by only four classes of taxa: Actinopterygii (RRA = 82.0%), Insecta (RRA = 11.3%), Arachnida (RRA = 5.4%) and Aconoidasida (RRA = 1.4%). Actinopterygii had the highest RRA across all prey communities of the farmed mahseer samples. However, the prey community of fish individuals ABI-A was dominated by Insecta whilst the gut digesta sample from fish FRI-C did not contain any Insecta, unlike the rest of farmed mahseer.

Fig. 4 shows the RRA values of species taxa that were successfully identified from the gut digesta of wild and farmed mahseer. The top 10 predominant prey species were *Cheumatopsyche charites, Ninguta schrenckii, Asyndetus* sp., *Chironomidae* sp., *Cantharis rustica, Potamanthus* sp., *Dolomedes plantarius, Cyprinus carpio, Plasmodium* sp. and *Ophiocytium majus*. The total RRA of these 10 taxa accounted for 60.5% of the prey abundance for all wild fish populations and 96.5% across the farmed fish population dataset (Supplementary Table S2).

A comparison of the prey community across the 22 gut digesta samples of mahseer revealed two species that were found in all samples, namely *C. carpio* and *Plasmodium* sp. Among these 22 samples, *C. carpio* exhibited a wider range of RRA (4.0% to 91.4%) compared to *Plasmodium* sp. (0.8% to 9.7%).

Several taxa were dominant but only to certain fish hosts, especially for fish captured from ROB. For instance, the abundance of *C. charites* was only demonstrated in the gut sample of ROB-A. Additionally, *Asyndetus* sp., *Potamanthus* sp. and *Chironomidae* sp. were identified in only one sample, involving different fish hosts from different river systems. At the individual level, ROB-A had the most diverse prey community with the highest relative abundance of “Others” with 43.8%, which consisted of 36 taxa. Whereas, EMP-B had the lowest relative abundance of “Others” with 1.6%, consisting of only six taxa. The species grouped under the “Others” category are listed in Supplementary Table S2.

Fish representing farmed populations originated from three different farming locations: FRI, ABI and TNB. Gut samples FRI-A and FRI-B were technical replications from the same host fish and served as control. The prey communities of farmed fish were predominated by three taxa, namely *C. carpio* (80.5%), *N. schrenckii* (8.3%) and *D. plantarius* (4.7%) (see Supplementary Table S2). Another eight taxa (*Deilephila porcellus, Apamea monoglypha, Zygaena filipendulae, Schizomida* sp., *Carassius auratus, Sinocyclocheilus rhinocerous, Oryzias latipes* and *Danio* sp.) showed average RRA values of less than 1%, ranging from 0.03% to 0.68%, and were grouped as “Others”. *C. carpio* dominated more than half of the prey community in all farmed fish samples, except for fish sample ABI-A, which was dominated exclusively by *N. schrenckii* (70%).

### Statistical Analyses

**FIGURE 5:**
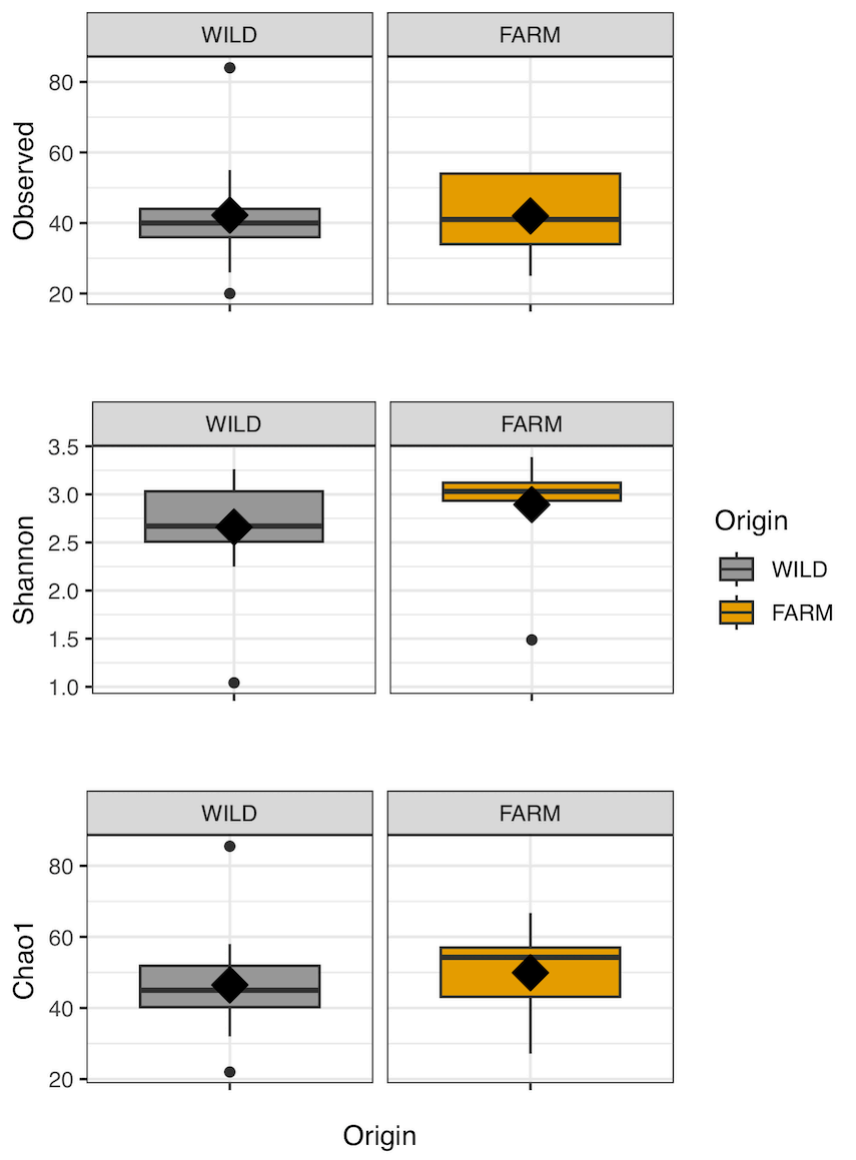
Boxplot of Alpha-diversity indices. Alpha diversity indexes are composite indexes reflecting abundance and consistency. The Shannon index reflects OTU diversity, while Chao1 indices reflect OTU abundance in samples. The greater the Shannon index, the higher the diversity of the prey community, and the greater the Chao index, the higher the expected species richness of the prey community. Diamond symbols represent the mean value for each indices calculated across all samples within the same origin.

Alpha diversity measures of the number of observed OTUs and Chao1 indices served as the measures of species richness, whereas the Shannon index was used to assess the evenness of the prey community in the gut (Fig. 5). A comparison of these indices among the fish populations from different origins (wild vs. farmed) revealed that the wild fish exhibited a slightly higher mean number of observed taxa (Obs_wild_ = 42.23) compared to the farmed mahseer (Obs_farmed_ = 42). Surprisingly, the Chao1 and Shannon indices for farmed mahseer depicted higher values (Chao_farmed_ = 49.9; Shannon_farmed_ = 2.89) than wild mahseer (Chao_wild_ = 46.5; Shannon_wild_ = 2.66), although the difference was not statistically significant.

**FIGURE 6:**
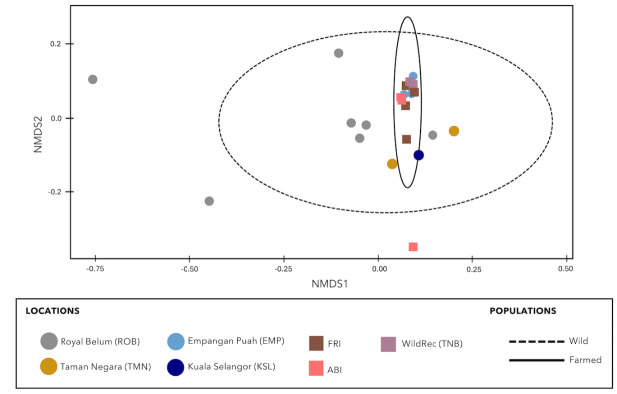
Non metric multidimensional scaling (NMDS) plot of beta diversity based on Bray-Curtis distance matrix (stress value = 0.1017). Each point represented the diet composition of individual mahseer sample while colours represent locations these samples were collected from. Samples with similar diet composition tended to be closer together, while points far apart from each other represent samples with dissimilar diet composition. Each ellipse represents a 95% confidence interval around each group.

Species turnover was assessed using the nonmetric multidimensional scaling (NMDS) analysis. As shown in Fig. 6, a stress value of less than 0.2 (stress value = 0.1017) indicates a “good fit” of the data. Prey community composition obtained from the gut content of each wild mahseer exhibited scattered distribution across the plot compared to farmed mahseer individuals that were clustered together. Fish from two wild locations (KSL and EMP) showed an overlap with the confidence ellipses of the farmed population. The NMDS analysis of turnover in prey community among mahseer locations rejected the null hypothesis of no difference in prey community diversity and taxonomic composition between wild and farmed fish (PERMANOVA test, pseudo-F = 1.9588, *p* = 0.0307).

Furthermore, the ANOSIM test, as shown in Table 2, identified the species responsible for significant differences observed between the wild and farmed mahseer populations in terms of their diet composition. *C. charites, A. sawyeri, P. astictus, E. albipennis, R. stictoptera, O. vitrina, R. punctulata, T. douglasi, A. vaga, L. closterocerca, S. ruficornis, Microtendipes* sp., *Antocha* sp., *L. monacha, D. saxonica, Mesostigmata* sp., *P. spinitarsis, Arrenurus* sp., *H. antarctica, Halicephalobus* sp. and *N. bachei* were among the species that contributed significant differences in the prey community between the wild and farmed mahseer populations.

## DISCUSSION

### Dietary Composition of Malaysian Mahseer (*Tor tambra*)

In this present study, the host COI amplicon has been taxonomically identified as *Tor tambra*, hence we classified our study species as *T. tambra*. Mahseers’ diet varies depending on the specific species. Past research suggested that they are mostly omnivorous and consume algae, submerged plants, insects, freshwater molluscs and snails and small fishes (Tan 1980; Dinesh *et al*. 2010). The findings of this study are consistent with those reported by previous studies (Tan 1980; Dinesh *et al*. 2010) whereby the *T. tambra*’s diet is diverse as their prey consists of Insecta, Arachnida (also assumed as insects), crustaceans (e.g., Ostracoda), Malacostraca, extracellular parasites, Aconoidasida and Actinopterygii. Malaysian mahseer is also regarded as a “bottom-feeder” in nature but can be trained to take the artificial floating in captivity (Ramezani-Fard & Kamarudin 2012). Previous research on feeding behaviour reported that *Tor tambroides* consumes fallen fruits from trees growing along riverbanks as part of its diet (Ambak *et al*. 2007; Siraj *et al*. 2007; Bami *et al*. 2017). However, this metabarcoding study did not assess the presence of plant-based materials in the mahseer’s GIT content. Future studies could explore this aspect using primer pairs specifically targeting plant-based dietary components. The presence of Insecta and Actinopterygii in this diet study is also consistent with the stomach content analysis of Tor putitora from the Mahakali River in Nepal by Mahaseth (2015), which revealed the presence of certain fish residues and insect body parts.

Depending on the river localities, the presence and high abundance of Insecta, Arachnida, and Actinopterygii in the fish GIT may be attributed to the switch from “bottom-feeder” to “open-water” eating habits. Mahseer species can get to the water’s surface to feed on fish and insects that inhabit in open water. The fish may also prey on eggs, fish larvae, larvae, pupae, adult insects and arachnids. Various species of Insecta, including caddisflies (Trichoptera), mayflies (Ephemeroptera), flies and mosquitoes (Diptera) and midges (Chironomidae) also appear as potential preys in a previous cyprinid diet study (Lammens & Hoogenboezem 1991; Piria *et al*. 2005). Aquatic insect species like Chironomids, Trichopteran, Ephemeropteran and Dipteran spend most of their entire life cycles on or near the water surface, making them a primary source of food for the fish (Nogales-Mérida *et al*. 2019). Some of the Actinopterygii species were also detected in the T. tambra digesta, mostly identified as relatively smaller fishes that support the diverse selection of fish diet for young mahseer, especially during the juvenile stage. Hence, the *T. tambra*’s feeding strategy enables them to eat a wide variety of aquatic prey and the species could be considered as a generalist.

Our findings highlight the challenges of using cytochrome oxidase I (COI) as a species tag for prey identification, particularly when analysing short sequence reads that may lead to ambiguous species assignments. While COI has been widely applied in characterising tropical ichthyofauna, its effectiveness can be influenced by the presence of cryptic species and gaps in genetic repositories (Meganathan *et al*. 2017). Additionally, primers designed for specific taxa may not always perform optimally across diverse ecosystems (Elbrecht & Leese 2017), which may explain our primer set – originally developed for freshwater macroinvertebrates – struggled to detect non-insect metazoans such as fish from Actinopterygii class. This limitation is particularly relevant when studying the *Cyprinidae* family, the largest and most widely distributed family of Malaysian freshwater fish, which dominates the natural habitat of mahseer. Given their abundance and adaptability (Zhao *et al*. 2020) to environmental changes (Bušová & Štancelová 2013), cyprinids are likely key dietary component, yet the high relative read abundance of *C. carpio* (commonly known as “Leekoh” and considered as invasive in the Malaysian natural riverine system) detected in the mahseer’s digesta suggest potentital misidentification of other cyprinids species. This highlight the need for improved regional reference libraries and refined primer sets to enhance taxonomic resolution.

The high abundance of Apicomplexan parasites identified as Plasmodium sp. was also detected among other prey eaten by mahseer. This intracellular eukaryote is known for thriving within other eukaryotic organisms and infecting a wide range of hosts from molluscs to mammals (Martínez-Occampo 2018). Thus, it is regarded as an important pathogen not only to humans but also to domestic animals and livestock with health and economic relevance worldwide. Recent studies have only identified mosquitoes, reptiles, birds, rodents and primates as the hosts of Plasmodium sp. (Miranda *et al*. 2022; Smith & Styczynski 2018). Given that this fish species is omnivorous, it is possible that Plasmodium larvae are engulfed by Tor tambra either in natural or artificial rearing.

All prey discovered in the farmed mahseer were also detected in the wild mahseer, with the exception of schizomids (Figs. 1 and 2). Although schizomids are frequently found in tropical areas, there is insufficient information to confirm their presence within freshwater bodies in Peninsular Malaysia. The presence of schizomids in the diet of farmed fish can be accounted to the use of imported fish pellets, which may contain schizomids that are unintentionally or intentionally included in the fish feed formula. It is worth to highlight that in understudied environments, such as freshwater in Malaysia, reference databases often lack sequences and corresponding taxonomic information for many organisms, thereby compromising the precision of prey assignation of this study (Cribdon *et al*. 2020; Hleap *et al*. 2021; Smith *et al*. 2022).

### Dietary Composition Variation Between Locations and Origins

This study initially hypothesised that prey diversity and richness consumed by wild fish would be higher than farm-origin fish. Yet, the alpha diversity indices depicted that mahseers’ prey community is not much diverse between wild and farm. Interestingly, the beta diversity analyses based on the NMDS ordination analysis and PERMANOVA demonstrated a significant difference in prey community variation between the samples across different sampling locations. The overlapping and grouping of the farmed mahseer individuals revealed that the structure of their prey community was more similar and resemblant to one another. On the contrary, the dietary composition of individual mahseer from the wild population showed a more scattered distribution across Axis I in the NMDS plot (Fig. 6), indicating that the prey communities within the GIT of wild *T. tambra* from different locations are more diverse and distinct from each other.

There are several contributing factors that can influence the differences in dietary diversity between wild and domestic *T. tambra*. The diversity and abundance of prey and predators are highly interdependent, resulting from dynamic population interactions (Doebeli *et al*. 2021). In the natural settings, the boom-bust dynamics in the ecosystem caused by prey-predator cycles are crucial for maintaining the stability of diversity and abundance of all species, thus explaining the high variability of prey selection by wild T. tambra. Besides population dynamics, natural and/or anthropogenic ecological disturbances may affect the stability and diversity of ecological systems (MacDougall et al. 2013). The pristine and highly protected jungle habitat provide a stable environment that supports the steady growth of the mahseer population. This ecosystem offer greater variability in prey availability for wild fish compared to artificial-reared fish.

It was also observed that some collected samples contained low GIT contents, indicating hunger or a prolonged period without food, especially for fish from the wild. This situation may explain the low alpha diversity indices among fish from the wild, which slightly differed than farmed fish. The diet composition also varies across individuals depending on their likelihood of successfully capturing prey at different times, or across different life stages usually reflected by their body size. Larger individuals may have access to a broader range of prey items or exhibit different feeding strategies compared to smaller individuals. On the other hand, cultured mahseer are usually raised in large numbers and in controlled environments where they are consistently fed with food pellets. However, any invertebrates or prey residing in or near the water source or fish ponds could potentially be food sources for them. Often, the water supply to the hatchery is filtered to reduce substances that may harm the aquaculture environment. These conditions can affect prey diversity and abundance, thereby explaining the low diversity and abundance of prey in farmed fish populations. Furthermore, uneaten food pellets, excretions and metabolic waste can alter the water composition in the aquaculture environment, such as oxygen and nitrogen concentrations, leading to changes in habitat suitability and subsequently causing loss or shifts in the abundance and diversity of native invertebrates (Boyd 2004; Uddin *et al*. 2016). Additionally, the farmers’ efforts to manage water quality to ensure the optimal growth and health of mahseer can also have similar effects on the occurrence and diversity of invertebrates.

All Trichoptera species (*C. charites, P. astictus* and *O. vitrina*) detected in the prey community samples are among the taxa that contribute to the significant differences in the diet composition between wild and farmed mahseer (Table 2). The differences in diet composition between these two populations may be influenced by environmental conditions. Compared to aquaculture centers, Trichoptera species tend to have greater abundance and diversity in their natural habitats. This is because these habitats are situated farther away from human activity centers and are characterised by a greater presence of vegetation (Hedberg 2019). The remaining invertebrates, consisting of 17 Diptera species, exhibit significant differences in the prey community of both populations. These invertebrates have the ability to adapt and thrive in almost every water body. The inclusion of these invertebrates in the diet of wild mahseer may be attributed to the natural prey preferences of these fish in their natural habitats.

### Conservation Implication and Aquaculture Insight

Food availability is crucial for the survival of fish in natural habitats and for optimal growth, fecundity, and productivity in captivity. This study found that the Malaysian mahseer appears to be an opportunistic generalist, feeding on readily available prey in its natural habitat. Despite being classified as a benthic feeder (Ramezani-Fard & Kamarudin 2012), the Malaysian mahseer also prefers insects, with a diet mainly comprising macroinvertebrates and some small fish. It was also revealed that T. tambra consumed at least 54 species from the Insecta, Actinopterygii, Ostracoda and Malacostraca classes, along with other aquatic organisms in smaller proportions.

The detailed dietary spectra identified in this study highlight the importance of prey composition in freshwater habitats for the thriving and long-term sustainability of wild mahseer populations. Conservation measures must protect the existence and abundance of these essential food sources. The overlap of certain prey taxa between wild and farm fish indicates that water sources also supply necessary aquatic communities in aquaculture settings. Investigating insect-based feed formulations for efficiently growing mahseer in captivity is essential as some fish species rely on insect larvae for protein during early life stages. Adopting streamside rearing facilities (Abdul Razak & Scribner 2021) using minimally treated water from nearby rivers can mimic physicochemical conditions for water supply and preserve aquatic communities as supplemental feed for fish, in addition to fish pellets.

Results from the high taxonomic resolution of DNA metabarcoding also denoted intraspecific variation in the diet of *T. tambra* across different localities, potentially reflecting the quality of riverine systems under proper habitat protection and management. Mahseer and their supporting habitats provide indicators of ecological function and societal benefits. Since mahseer are mainly restricted to upper river streams and are sensitive to environmental disturbances, preserving well-maintained habitats is vital for providing suitable food sources for mahseer and other aquatic species (Jaafar *et al*. 2021). The extent of human activities near the sampled rivers indirectly influenced the number of fish collected from each location, highlighting variations in mahseer habitat quality.

DNA metabarcoding can summarise mahseer’s dietary composition and feeding ecology, benefiting aquaculture efforts and informing conservation strategies. Moreover, the findings of this study can provide fundamental information to bridge the knowledge gap regarding mahseer species, particularly in terms of their diet, ultimately aiding the efforts to understand and conserve mahseer populations in their natural habitats. This diet characterisation can also contribute to the formulation of effective feed pellets for aquaculture purposes as a mitigation measure to alleviate pressure on wild mahseer populations.

## SUPPLEMENTARY MATERIALS

**TABLE S1:** Percentage of Frequency of Occurrence (FOO), Relative Read Abundance (RRA), and Weighted Percentage of Occurrence (wPOO) at the Class level among wild and farmed mahseer.

Class	FOO (%)			RRA (%)			wPOO (%)		

	Overall	Wild	Farmed	Overall	Wild	Farmed	Overall	Wild	Farmed

	*n* = 22	*n* = 13	*n* = 9	*n* = 22	*n* = 13	*n* = 9	*n* = 22	*n* = 13	*n* = 9
Insecta	95.45	100.00	88.89	41.43	48.65	11.26	18.42	16.46	22.86
Arachnida	95.45	92.31	100.00	9.09	9.98	5.36	18.42	15.19	25.71
Ostracoda	4.55	7.69	0.00	0.42	0.52	0.00	0.88	1.27	0.00
Malacostraca	4.55	7.69	0.00	0.15	0.19	0.00	0.88	1.27	0.00
Actinopterygii	100.00	100.00	100.00	34.11	22.65	81.97	19.30	16.46	25.71
Chromadorea	40.91	69.23	0.00	2.32	2.88	0.00	7.89	11.39	0.00
Bdelloidea	4.55	7.69	0.00	0.37	0.45	0.00	0.88	1.27	0.00
Monogononta	4.55	7.69	0.00	0.17	0.22	0.00	0.88	1.27	0.00
Aconoidasida	100.00	100.00	100.00	6.95	8.27	1.41	19.30	16.46	25.71
Hydrozoa	9.09	15.38	0.00	0.71	0.88	0.00	1.75	2.53	0.00
Phaeophyceae	18.18	30.77	0.00	0.50	0.62	0.00	3.51	5.06	0.00
Xanthophyceae	40.91	69.23	0.00	3.79	4.70	0.00	7.89	11.39	0.00

Total				100.00	100.00	100.00	100.00	100.00	100.00

**TABLE S2:** Percentage of Frequency of Occurrence (FOO), Relative Read Abundance (RRA), and Weighted Percentage of Occurrence (wPOO) at the Species level among wild and farmed mahseer.

Phylum	Class	Identity at the lowest taxonomy level (Species)	Common name	FOO (%)			wPOO (%)			RRA (%)		

				Overall	Wild	Farmed	Overall	Wild	Farmed	Overall	Wild	Farmed

				*n* = 22	*n* = 13	*n* = 9	*n* = 22	*n* = 13	*n* = 9	*n* = 22	*n* = 13	*n* = 9
Arthropoda	Insecta	*Cheumatopsyche charites*	Caddisflies	4.55	7.69	0.00	0.41	0.58	0.00	19.99	24.78	0.00
	Insecta	*Polymorphanisus astictus*	Caddisflies	4.55	7.69	0.00	0.41	0.58	0.00	2.10	2.60	0.00
Arthropoda	Insecta	*Oestropsyche vitrina*	Caddisflies	4.55	7.69	0.00	0.41	0.58	0.00	1.01	1.26	0.00
Arthropoda	Insecta	*Anopheles sawyeri*	Mosquitos	18.18	30.77	0.00	1.64	2.33	0.00	4.20	5.21	0.00
Arthropoda	Insecta	*Panorpa liui*	Scorpionflies	9.09	15.38	0.00	0.82	1.16	0.00	2.47	3.06	0.00
Arthropoda	Insecta	*Potamanthus* sp.	Mayflies	4.55	7.69	0.00	0.41	0.58	0.00	0.45	0.55	0.00
Arthropoda	Insecta	*Ephemera nadinae*	Mayflies	13.64	23.08	0.00	1.23	1.74	0.00	0.35	0.43	0.00
Arthropoda	Insecta	*Callibaetis pretiosus*	Mayflies	13.64	23.08	0.00	1.23	1.74	0.00	0.26	0.32	0.00
Arthropoda	Insecta	*Ninguta schrenckii*	Butterflies	9.09	7.69	7.69	0.82	0.58	1.39	1.67	0.08	8.29
Arthropoda	Insecta	*Endochironomus albipennis*	Flies	4.55	7.69	0.00	0.41	0.58	0.00	1.54	1.91	0.00
Arthropoda	Insecta	*Riethia stictoptera*	Flies	4.55	7.69	0.00	0.41	0.58	0.00	1.40	1.74	0.00
Arthropoda	Insecta	*Asyndetus* sp.	Flies	9.09	15.38	0.00	0.82	1.16	0.00	0.89	1.10	0.00
Arthropoda	Insecta	*Rhinoleucophenga punctulata*	Flies	4.55	7.69	0.00	0.41	0.58	0.00	0.80	0.99	0.00
Arthropoda	Insecta	*Sarcophaga ruficornis*	Flies	4.55	7.69	0.00	0.41	0.58	0.00	0.44	0.55	0.00
Arthropoda	Insecta	*Microtendipes* sp.	Flies	4.55	7.69	0.00	0.41	0.58	0.00	0.29	0.36	0.00
Arthropoda	Insecta	*Pagastia* aff *. Lanceolata*	Flies	9.09	15.38	0.00	0.82	1.16	0.00	0.17	0.22	0.00
Arthropoda	Insecta	*Atherigona oryzae*	Flies	4.55	7.69	0.00	0.41	0.58	0.00	0.12	0.14	0.00
Arthropoda	Insecta	*Antocha* sp.	Flies	4.55	7.69	0.00	0.41	0.58	0.00	0.11	0.13	0.00
Arthropoda	Insecta	*Coenosia* sp.	Flies	4.55	7.69	0.00	0.41	0.58	0.00	0.05	0.07	0.00
Arthropoda	Insecta	*Timema douglasi*	Walking sticks	4.55	7.69	0.00	0.41	0.58	0.00	0.52	0.64	0.00
Arthropoda	Insecta	*Cantharis rustica*	Beetles	59.09	61.54	38.46	5.33	4.65	6.94	0.54	0.31	1.54
Arthropoda	Insecta	*Ceratopogonidae* sp.	Biting midges	9.09	15.38	0.00	0.82	1.16	0.00	0.50	0.62	0.00
Arthropoda	Insecta	*Chironomidae* sp.	Nonbiting midges	9.09	15.38	0.00	0.82	1.16	0.00	0.53	0.65	0.00
Arthropoda	Insecta	*Deilephila porcellus*	Moths	50.00	53.85	30.77	4.51	4.07	5.56	0.46	0.41	0.68
Arthropoda	Insecta	*Apamea monoglypha*	Moths	59.09	61.54	38.46	5.33	4.65	6.94	0.25	0.18	0.57
Arthropoda	Insecta	*Zygaena filipendulae*	Moths	27.27	23.08	23.08	2.46	1.74	4.17	0.05	0.03	0.17
Arthropoda	Insecta	*Lymantria monacha*	Black-arched tussock moth	4.55	7.69	0.00	0.41	0.58	0.00	0.07	0.09	0.00
Arthropoda	Insecta	*Bombus sylvestris*	Bees	27.27	46.15	0.00	2.46	3.49	0.00	0.10	0.13	0.00
Arthropoda	Insecta	*Dolichovespula saxonica*	Wasps	4.55	7.69	0.00	0.41	0.58	0.00	0.07	0.09	0.00
Arthropoda	Arachnida	*Clitaetra* sp.	Spiders	4.55	7.69	0.00	0.41	0.58	0.00	0.02	n.02	n.00
Arthropoda	Arachnida	*Dolomedes plantarius*	Spiders	95.45	92.31	69.23	8.61	6.98	12.50	1.64	0.91	4.70
Arthropoda	Arachnida	*Philodromus spinitarsis*	Spiders	4.55	7.69	0.00	0.41	0.58	0.00	0.08	0.10	0.00
Arthropoda	Arachnida	*Schizomida* sp.	Arachnids	9.09	0.00	15.38	0.82	0.00	2.78	0.13	0.00	0.66
Arthropoda	Arachnida	*Joubertophyllodes ampulaceus*	Mites and ticks	4.55	7.69	0.00	0.41	0.58	0.00	0.04	0.05	0.00
Arthropoda	Arachnida	*Mesostigmata* sp.	Mites and ticks	4.55	7.69	0.00	0.41	0.58	0.00	4.79	5.94	0.00
Arthropoda	Arachnida	*Lebertia* sp.	Mites and ticks	9.09	15.38	0.00	0.82	1.16	0.00	2.13	2.64	0.00
Arthropoda	Arachnida	*Arrenurus* sp.	Mites and ticks	4.55	7.69	0.00	0.41	0.58	0.00	0.25	0.31	0.00
Arthropoda	Ostracoda	*Eucypris virens*	Crustaceans	4.55	7.69	0.00	0.41	0.58	0.00	0.42	0.52	0.00
Arthropoda	Malacostraca	*Hyperiella antarctica*	Amphipods	4.55	7.69	0.00	0.41	0.58	0.00	0.15	0.19	0.00
Chordata	Actinopterygii	*Cyprinus carpio*	Common carp	100.00	100.00	69.23	9.02	7.56	12.50	33.49	22.24	80.52
Chordata	Actinopterygii	*Carassius auratus*	Goldfish	72.73	69.23	53.85	6.56	5.23	9.72	0.20	0.13	0.49
Chordata	Actinopterygii	*Sinocyclocheilus rhinocerous*	Rhinoceros golden-line barbel	72.73	69.23	53.85	6.56	5.23	9.72	0.24	0.15	0.58
Chordata	Actinopterygii	*Oryzias latipes*	Japanese rice fish	50.00	23.08	61.54	4.51	1.74	11.11	0.16	0.12	0.34
Chordata	Actinopterygii	*Danio* sp.	Zebrafish	27.27	23.08	23.08	2.46	1.74	4.17	0.02	0.02	0.03
Nematoda	Chromadorea	*Ficophagus* cf *. centerae*	Nematodes	13.64	23.08	0.00	1.23	1.74	0.00	1.01	1.25	0.00
Nematoda	Chromadorea	*Halicephalobus* sp.	Nematodes	22.73	38.46	0.00	2.05	2.91	0.00	1.03	1.28	0.00
Nematoda	Chromadorea	*Nematodirus oiratianus*	Nematodes	22.73	38.46	0.00	2.05	2.91	0.00	0.28	0.35	0.00
Rotifera	Bdelloidea	*Adineta vaga*	Rotifers	4.55	7.69	0.00	0.41	0.58	0.00	0.37	0.45	0.00
Rotifera	Monogononta	*Lecane closterocerca*	Rotifers	4.55	7.69	0.00	0.41	0.58	0.00	0.17	0.22	0.00
Apicomplexa	Aconoidasida	*Plasmodium* sp.	Apicomplexans	100.00	100.00	69.23	9.02	7.56	12.50	6.95	8.27	1.41
Cnidaria	Hydrozoa	*Nemopsis bachei*	Hydrozoans	9.09	15.38	0.00	0.82	1.16	0.00	0.71	0.88	0.00
Ochrophyta	Phaeophyceae	*Petalonia filiformis*	Brown algae	13.64	23.08	0.00	1.23	1.74	0.00	0.35	0.43	0.00
Ochrophyta	Phaeophyceae	*Vimineoleathesia japonica*	Brown algae	18.18	30.77	0.00	1.64	2.33	0.00	0.15	0.18	0.00
Ochrophyta	Xanthophyceae	*Ophiocytium majus*	Yellow-green algae	40.91	69.23	0.00	3.69	5.23	0.00	3.79	4.70	0.00

## Figures and Tables

**TABLE 1:** Distribution of samples across different populations and locations.

Origins	Locations	Abbreviation	Number of samples
Wild	Sungai Tiang, Royal Belum, Perak	ROB	7
	Sungai Keniam, Taman Negara, Pahang	TMN	2
	Sungai Puah, Terengganu	EMP	3
	Kuala Selangor, Selangor	KSL	1
Farmed	Agro-Biotechnology Institute, Selangor	ABI	3
	Fisheries Research Institute, Negeri Sembilan	FRI	3
	WildRec TNB, Empangan Puah, Terengganu	TNB	2

Notes: The “Origins” column indicates whether the samples were obtained from wild or farmed sources. The “Locations” column lists the specific locations where the samples were collected. The “Number of samples” column displays the total number of samples collected from each location under each population category.

**TABLE 2:** ANOSIM test conducted on the prey community in the GI samples between wild and farmed mahseer populations. Taxa or species marked with an asterisk (*) represent taxonomic groups that contribute to significant differences between the two populations (*p* < 0.05).

Taxa	*p*-values	Taxa	p-values
C. charites*	0.0451	D. saxonica*	0.0449
A. sawyeri*	0.0397	*Coenosia* sp.	0.5851
*P. liui*	0.0776	*J. ampulaceus*	0.589
P. astictus*	0.0484	*Clitaetra* sp.	0.454
*N. schrenckii*	0.2397	*Mesostigmata* sp.*	0.0458
E. albipennis*	0.0471	*Lebertia* sp.	0.0792
R. stictoptera*	0.0452	*D. plantarius*	0.0669
*Asyndetus* sp.	0.2001	*Schizomida* sp.	0.1497
O. vitrina*	0.0466	P. spinitarsis*	0.0459
R. punctulata*	0.0429	*Arrenurus* sp.*	0.0456
T. douglasi*	0.045	*E. virens*	0.6867
*Chironomidae* sp.	0.545	H. antarctica*	0.0464
*C. rustica*	0.5125	*C. auratus*	0.2212
*Potamanthus* sp.	0.5029	*S. rhinocerous*	0.7163
*Ceratopogonidae* sp.	0.0784	*O. latipes*	0.7796
*D. porcellus*	0.1263	*D. rerio*	0.0755
S. ruficornis*	0.0414	*C. carpio*	-
*E. nadinae*	0.1976	L. closterocerca*	0.046
*A. monoglypha*	0.6938	*Halicephalobus* sp.*	0.0367
*Microtendipes* sp.*	0.0471	*N. oiratianus*	0.1013
*C. pretiosus*	0.1437	A. vaga*	0.046
*Pagastia aff. Lanceolata*	0.0654	*Ficophagus cf. centerae*	0.1317
*A. oryzae*	0.3651	*Plasmodium* sp.	0.1409
*Antocha* sp.*	0.0451	N. bachei*	0.004
*B. sylvestris*	0.2243	*P. filiformis*	0.1756
L. monacha*	0.051	*V. japonica*	0.2463
*Z. filipendulae*	0.1835	*O. majus*	0.1534

## Data Availability

Supplementary Tables 1 and 2 for FOO, wPOO and RRA were included as supplementary materials.
